# Supplementary material for: The molecular dynamics of subdistal appendages in multi-ciliated cells
Source: Nat Commun. 2021 Jan 27;12:612. doi: 10.1038/s41467-021-20902-4 (PMC7840914; doi:10.1038/s41467-021-20902-4)
Supplement: Supplementary file 1 — Supplementary Information [file 41467_2021_20902_MOESM1_ESM.pdf]

Supplementary Information

The molecular dynamics of subdistal appendages in multi-ciliated cells

Ryu et al. Supplementary Information includes Supplementary Method, Supplementary Table and seven Supplementary Figures with legnds

Supplementary methods

Analysis of translational polarity. For translational polarity analysis, we used Fiji software to draw vectors from the centers of apical surfaces of each cell to the centers of BB patches or to the cell perimeters in the same direction of BB patch vector. BB patch displacement was analyzed by dividing the magnitude of BB patch vector by the length of the line drawn from the center of the cell surface to the cell perimeter. For tissue-wide translational polarity analysis, the angles of each BB patch vector were obtained from approximately 8 cells in the high power field (64.31 x 64.31 μm^2), averaged for the area, normalized to 225° and then their distribution was plotted on a histogram.

Real-time quantitative RT-PCR analysis. Total RNAs from dissected LWs was isolated using TRIzol reagent (Invitrogen, 15596-026). cDNA was synthesized using up to 4 μg of total RNA and the QIAGEN OneStep RT-PCR Kit (QIAGEN, 210210) in 80 μl 1X QIAGEN OneStep RT-PCR buffer containing 400 μM of each dNTP, 0.5 μM oligodT, 5 units of RNase inhibitor, and 1 μl Omniscript Reverse Transcriptase. Gene expression was detected by real-time quantitative RT-PCR in a LightCycler 96 Instrument (Roche, 05815916001) using qPCRBIO SyGreen Blue Mix (PCR Biosystems, PB20.15-05). The PCR amplification primers for *KI67, FOXJ1, GEMC1, MCIDAS, DEUP1, CEP152, VANGL1, VANGL2, FZD3, CELSR1, CELSR2, CELSR3, ANKS1A, ANKS1B,* and *GAPDH* mouse genes are described in the supplementary table. mRNA expression levels were normalized to *GAPDH* mRNA expression.

Magnetic resonance microimaging. Mice were anesthetized with a mixture of air and 3% isoflurane (Hana Pharm. Co., Ltd., Kyonggi-Do, Korea) and then placed in a MRI-compatible animal cradle. The isoflurane concentration was maintained at 1.5 ± 0.5%, adjusted to maintain the animal at 100 ± 20 breaths/min throughout the MRI sessions. Respiratory signals and body temperature were monitored using a physiological monitoring system (SA Instruments, Inc., Stony Brook, NY, USA). Body temperature was maintained by circulating water through heating pads. MRI was conducted using a 9.4 T horizontal-bore Bruker Avance III HD imaging system (Bruker Biospin, Ettlingen, Germany) and a 23-mm diameter transmit/receive volume radio frequency (RF) coil (Bruker Biospin, Ettlingen, Germany). An axial T2 weighted turbo rapid acquisition with relaxation enhancement (RARE) image (T2 WI: repetition time [TR] = 4300 ms, echo time [TE] = 26 ms, number of averages [NA] = 5, field of view [FOV] = 20 mm x 20 mm, matrix size = 256 x 256, slice thickness = 250 μm, number of slices = 5, RARE factor = 8, and acquisition time = 11 min 28 sec) was acquired around the brain. 3D-projection image reconstruction and ventricle volume measurement were processed by Fiji software. Twenty consecutive coronal images were merged into a single picture for each brain. Ventricles appeared bright on T2-weighted μMRI mouse brain images are shown in gray scale and color LUT (Look Up Table) display. Color-coded images display brain ventricles with anterior-posterior (A-P) direction. Red colors represent an anterior region of the brain ventricle, while blue colors represent a posterior region.

Supplementary table

Primary antibodies

Name	Isotype		Reference		Usage
ZO-1-488	Mouse	IgG1	Invitrogen	339188	IHC
α-Tubulin	Mouse	IgG2a	Santa Cruz	SC5286	IHC
GT335	Mouse	IgG1	Adipogen	AG-20B-0020	IHC
Ac-Tubulin	Mouse	IgG2b	Sigma-aldrich	T7451	IHC
γ-Tubulin	Mouse	IgG1	Abcam	ab11316	IHC
CEP164	Rabbit	IgG	Atlas antibodies	HPA037606	IHC
CEP19	Rabbit	IgG	Proteintech	26036-1-AP	IHC, WB, IP
CEP350	Rabbit	IgG	Novus	NB100-59811	WB, IP
ODF2	Rabbit	IgG	Atlas antibodies	HPA001874	IHC
CNTRL	Mouse	IgG1	Santa cruz	SC-365521	IHC
FOP	Mouse	IgG2b	Abnova	H00011116-M01	IHC, WB, IP
Centrin	Mouse	IgG2a	Millipore	04-1624	IHC
Frizzled3	Mouse	IgG2a	Sigma-aldrich	WH0007976M9	IHC
Vangl1	Rabbit	IgG	Atlas antibodies	HPA025235	IHC
ANKS1A	Rabbit	IgG	Bethyl	A303-049A	IHC
ANKS1A	Rabbit	IgG	Bethyl	A303-050A	WB, IP
GFP	Chick	IgY	Abcam	ab13970	IHC

Secondary antibodies

Name	Reference		Dilution
Goat anti-mouse IgG1 Alexa 488	Invitrogen	A21121	1/800
Goat anti-mouse IgG2b Alexa488	Invitrogen	A21141	1/800
Goat anti-mouse IgG1 Alexa568	Invitrogen	A21124	1/800
Goat anti-mouse IgG2a Alexa568	Invitrogen	A21134	1/800
Goat anti-mouse IgG2b Alexa568	Invitrogen	A21144	1/800
Donkey anti-rabbit IgG Alexa488	Invitrogen	A21206	1/800
Goat anti-rabbit IgG Alexa568	Invitrogen	A11036	1/800
Goat anti-rabbit IgG Alexa647	Invitrogen	A21244	1/800
Goat anti-chick IgY Alexa647	Invitrogen	A32933	1/800
Goat anti-Mouse IgG (H+L) HRP	Sigma-aldrich	A4416	1/1000
Goat anti-Rabbit IgG (H+L) HRP	Invitrogen	G-21234	1/1000

Supplementary table 1.

Oligonucleotides	Name		Sequence (5' → 3')	Size
	ANKS1A conventional KO	Forward 1	TGAAGGCACATGAGGCTGAG	20 mer
		Reverse	ACAGCGTTTGCATCTTGCTG	20 mer
		Forward 2	ATGTCATAGCTGTTTCCTGT	20 mer
	ANKS1A inducible KO	Forward	GATTCCTGGAAGCCCGAGAAGAACT	25 mer
		Reverse	TTGAACTCACAGAGCTGTCTGCCTG	25 mer
	YFP	Forward	GGGAGGGGAGTGTTGCAATACCTTT	25 mer
		Reverse	AAGACCGCGAAGAGTTTGTC	20 mer
	ANKS1A-CreER	Forward	TGGATAGTGAAACAGGGGCAATGGT	25 mer
		Reverse	TCTCCACCATGCCCTCTACACATTT	25 mer
	Morpholino	*xanks1a*-MO	AATAACTCCTGTTCTTTCCCCATCC	25 mer
	KI67	Forward	CCTGCCCGACCCTACAAAAT	20 mer
		Reverse	TGCTGCTTCTCCTTCACTGG	20 mer
	FOXJ1	Forward	GACGTGGACTATGCCACCAA	20 mer
		Reverse	ATGGAATTCTGCCAGGTGGG	20 mer
	GEMC1	Forward	ACCTCTCATCCCAGAGCAGT	20 mer
		Reverse	TGAGCCATGTGTTCCCTGAC	20 mer
	MCIDAS	Forward	CTCCCCTGACCAACAGTGAC	20 mer
		Reverse	TCTTTCAGCTGCACGTTCCT	20 mer
	DEUP1	Forward	ATATCCTTCCGGGCTTCCCT	20 mer
		Reverse	GGTGTGTCTCTGCAGCTCAT	20 mer
	CEP152	Forward	GAAGGACCACGTGCAACAAC	20 mer
		Reverse	TCCTGCACAGAGCCTGATTG	20 mer
	VANGL1	Forward	CTCACCCCCATTGCCTTCAT	20 mer
		Reverse	TGCTTGCGGAAGAAAAGTGC	20 mer
	VANGL2	Forward	AAGCTGCTCATCCTGCTGTT	20 mer
		Reverse	AAGATGCGCACACCGTAGAA	20 mer
	FZD3	Forward	GAATCAGGTCTGGACGACTCA	21 mer
		Reverse	CATCTGGGAGACAACATGGA	20 mer
	CELSR1	Forward	TCTCAGAGGAACTTCTGCGATG	22 mer
		Reverse	CAGGTACCAAGGCACAGAGATG	22 mer
	CELSR2	Forward	CCAGTGGCCTTTGCTGTTTC	20 mer
		Reverse	TTGACAGAGAGCAGTGCCAG	20 mer
	CELSR3	Forward	GGTCAGGGTCCAGGGTATCT	20 mer
		Reverse	TCCACTCTTTTGCGGATGCT	20 mer
	ANKS1A	Forward	CAGGCATTTGAGGTGGCCTA	20 mer
		Reverse	GGCACTGCAGACTTCCTCAT	20 mer
	ANKS1B	Forward	AAAGGGGAGTGCTCTTCACG	20 mer
		Reverse	CTTCCTGCGCCTTCCAAGTA	20 mer
	GAPDH	Forward	AGGTCGGTGTGAACGGATTTG	21 mer
		Reverse	TGTAGACCATGTAGTTGAGGTCA	23 mer
	anks1a	Forward	GTCAGATCTCGTCACACTGC	20 mer
		Reverse	AGACTTGGACAGACTCCTGC	20 mer
	odc1	Forward	CCCTATAAGACAAGGAATAC	20 mer
		Reverse	TCCATTCCGCTCTCCTGAGCAC	22 mer

Supplementary table 2.

Softwares	Name	Reference
	Fiji (1.52p)	https://fiji.sc/
	SigmaPlot (7.0)	Systat Software Inc.
	Prism Graphpad (5.01)	GraphPad software
	ZEN blue (1.1.2.0)	Carl Zeiss Microscopy
	ZEN black (8.1)	Carl Zeiss Microscopy
	Photoshop (7.0.1)	Adobe
	Premiere Pro 2019	Adobe

Supplementary table 3.

Supplementary figures

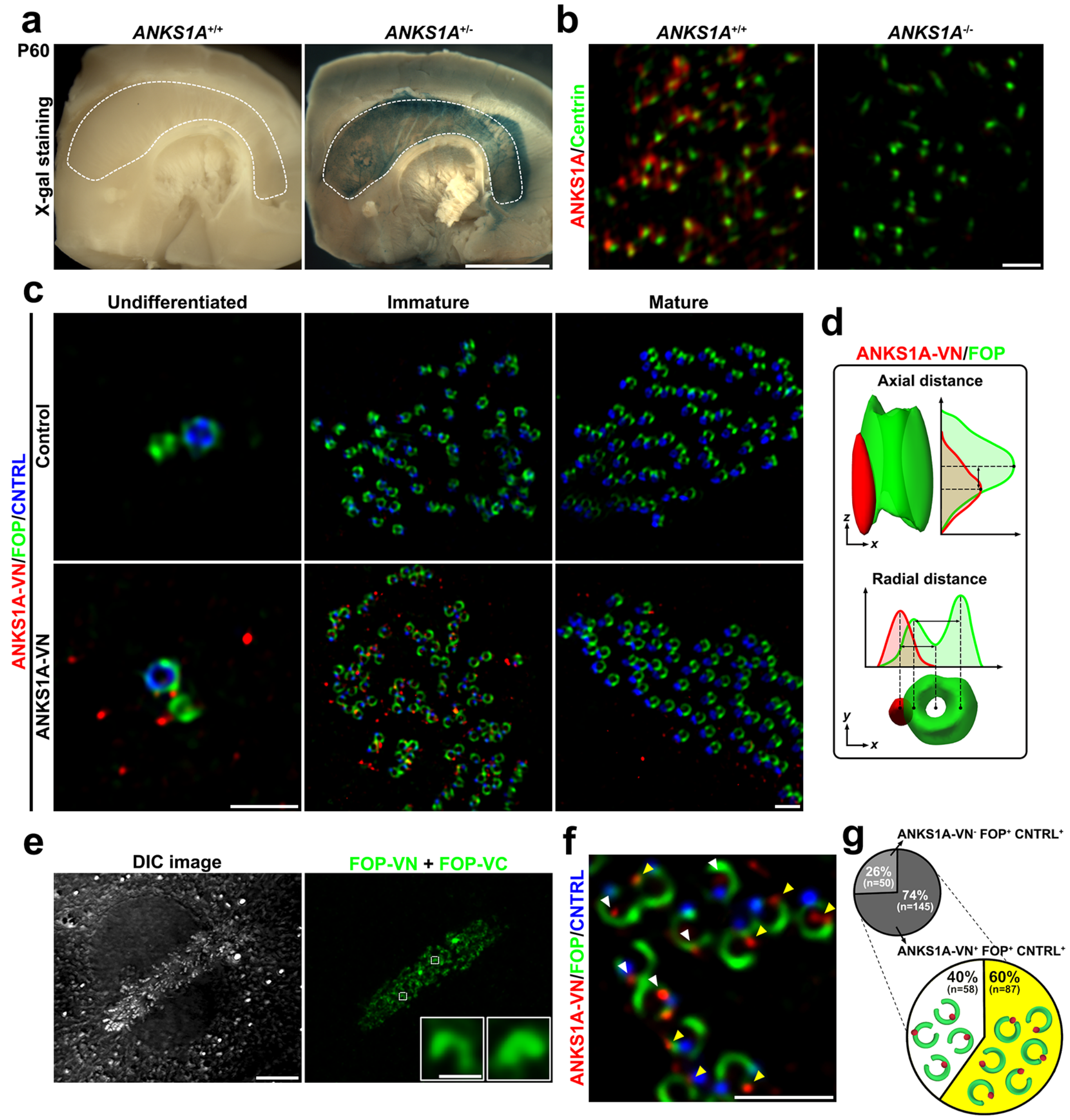

Supplementary Figure 1. (a) LWs (marked by dotted lines) with other forebrain tissues were subjected to X-gal staining. Scale bar, 500 μm. (b) Primary E1 cells were cultured for 8 days after serum starvation; centrin, a BB marker. Scale var, 1 μm. (c) 3DSIM micrographs of E1 cell BBs representing three different developmental stages. The LWs at P4 (undifferentiated and immature) or P21 (mature) were co-stained with FOP, CNTRL, and GFP antibodies. In immature cells, most of centrioles were localized to the apical surface but those apically docked BBs displayed a random orientation of the FOP-negative regions. In contrast, in mature cells, almost all of apically docked BBs display a uniform alignment of the FOP-negative regions. Scale bars, 1 μm. (d) Linear 3D SIM measurement analysis. FOP was used as a reference in each experiment for both axial and radial distances of other BB proteins. (e) Bimolecular fluorescent complementation analysis. The N-terminal (VN) and C-terminal (VC) portions of GFP were fused with the C-terminus of FOP, respectively. These constructs were transfected into primary E1 progenitors and then the differentiated cells with multi-cilia (as shown in DIC image) were further analyzed by Confocal microscopy. Scale bar, 5 μm (left) and 500 nm (right). (f and g) The co-localization of ANKS1A puncta in SDAs (shown in Fig. 1k) is illustrated in an enlarged picture (f) with a diagram showing their distribution (g). Scale bar, 1 μm. The yellow arrowheads mark the ANKS1A puncta in regions of reduced FOP staining whereas the white arrowheads point to the ANKS1A puncta in FOP-stained regions. N indicates the number of BBs.

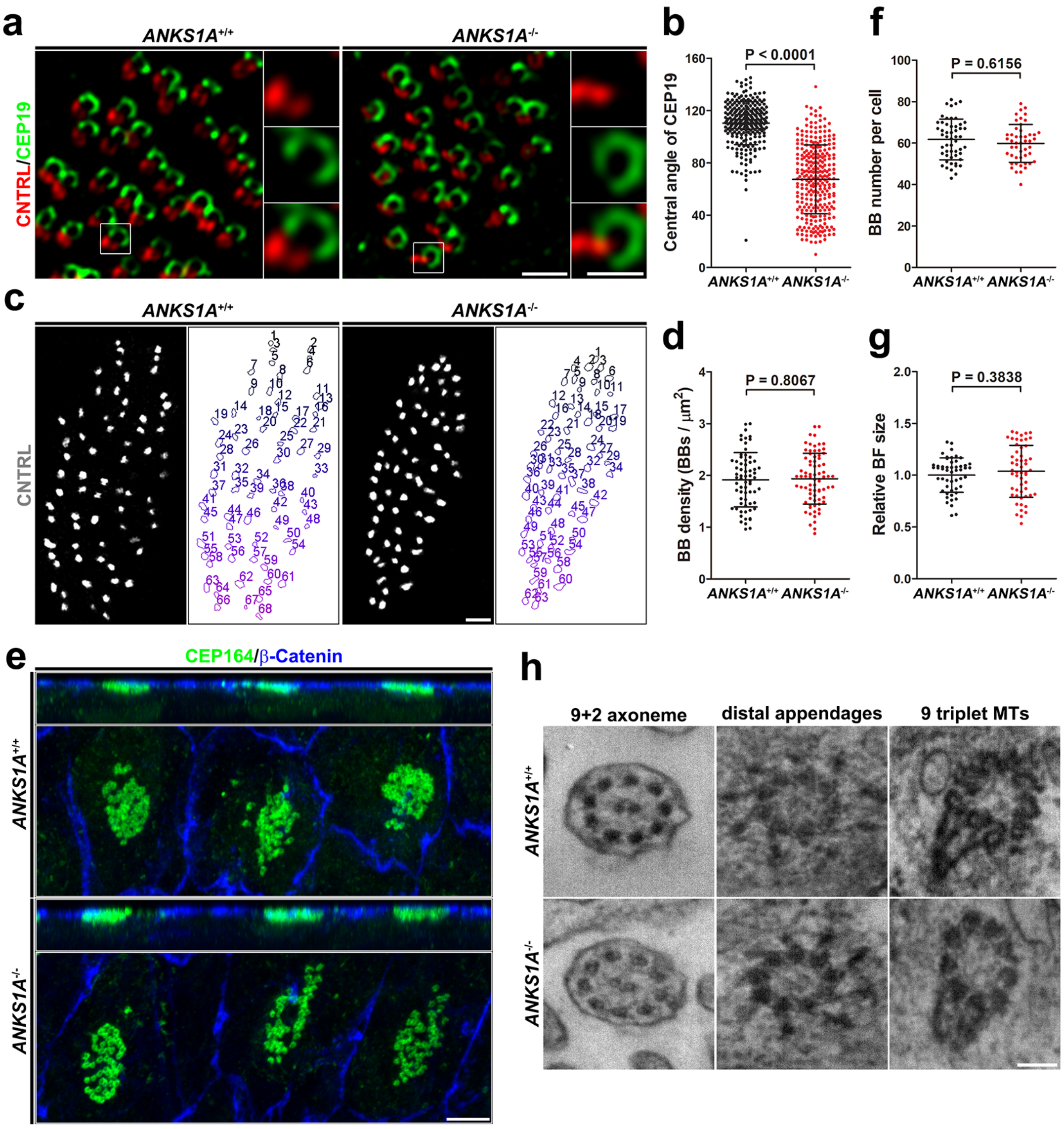

Supplementary Figure 2. (a) Experiments were performed as described in Fig. 2c, except that CEP19 was used instead of FOP. (b) The data in panel a were quantified. The data represent means ± SD. Each point on the graph represents a central angle of a CEP19-negative region. *ANKS1A*$^{+/+}$ set, n = 254 BBs; *ANKS1A*$^{-/-}$, n = 285 BBs. (c and d) 3D object count maps were generated with the three-dimensional object counter plugin in the Fiji software package. Each outline indicates a CNTRL-positive focus that can be used for assessing the number of BBs but also the BB density in a single cell. Scale bar for a and c, 1 µm. (e and f) Both apical surface docking of BBs and their number in each cell were analyzed using CEP164 staining. Note that the BB density based on CEP164 staining was not significantly different between WT and KO samples (data not shown). Scale bar, 5 µm. (g) The data represent means ± SD. Each point on the graph represents the overall size of a BF based on a serial TEM analysis as shown in Fig 2h. *ANKS1A*$^{+/+}$ set, n = 51 BBs; *ANKS1A*$^{-/-}$, n = 54 BBs. (h) Thin section images of ciliary axoneme, distal appendages, and nine triplet microtubules (MTs) in the LWs for young WT and KO mice at P45. Note that we scrutinized approximately 100 basal bodies from WT or KO samples, indicating that the 9+2 ciliary MT structure, distal appendage and triplet MT structure were not disturbed in KO. Scale bar, 100 nm.

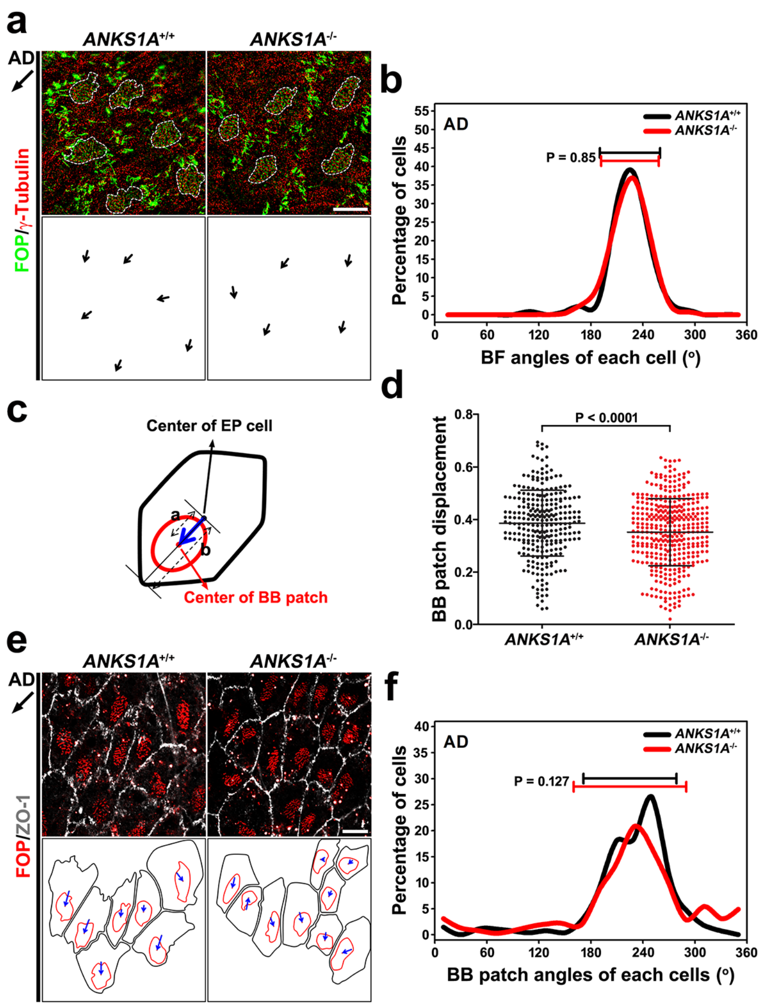

Supplementary Figure 3. (a) Each BB cluster is outlined with a dotted line. Black arrows indicate the average BF angle in each cell. (b) Histogram showing the distribution of the average angle of the vectors shown in panel a. Error bars on the graph represent CSD. (c) Schematic showing BB patch vector angle (marked by a blue arrow) and BB patch displacement (measured as a/b). (d) BB patch displacement was slightly but significantly affected in *ANKS1A* KO mice. The data represent means ± SD. Each point on the graph is the BB patch displacement of an individual E1 cell. *ANKS1A*$^{+/+}$ set, n = 275 cells from 3 mice; *ANKS1A*$^{-/-}$, n = 389 cells from 3 mice. (e) Immunohistochemical staining of ZO-1 (white) and FOP (red) on mouse LWs (top panels). ZO-1 delineates the cell boundaries (outlined in black), while FOP marks the BB patches (outlined in red) on the apical surface. Blue arrows in the bottom panels were drawn from the center of each cell to the cell's BB patch. Scale bar for a and e, 10 μm. (f) Histogram showing the distribution of BB patch angles in *ANKS1A* WT and KO samples. Error bars on the graph represent CSD. *ANKS1A*$^{+/+}$ set, n = 275 cells from 3 mice; *ANKS1A*$^{-/-}$, n = 389 cells from 3 mice.

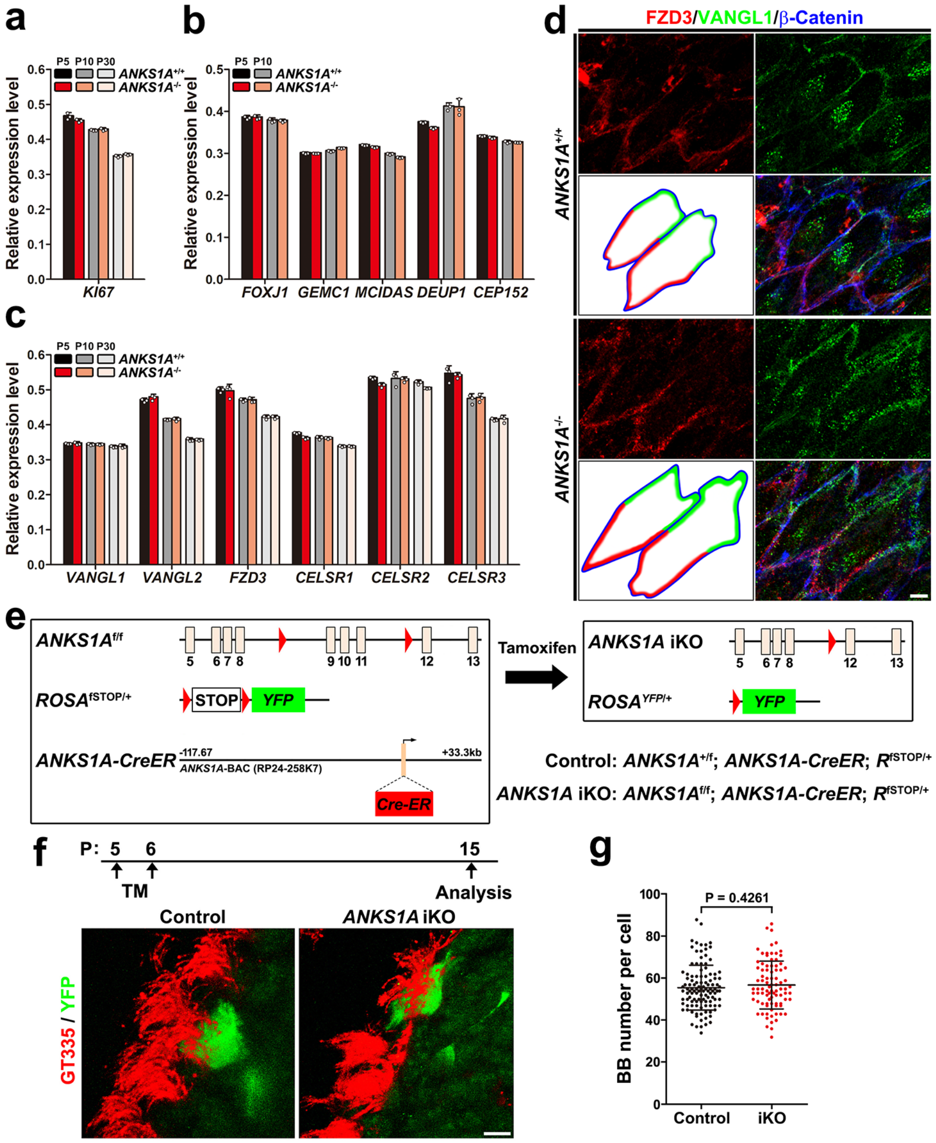

Supplementary Figure 4. (a-c) qRT-PCR analysis using total RNAs extracted from the LWs at the indicated postnatal stages. (d) Asymmetric localization of VANGL1 and FZD3 in ependymal cells. We found that antibodies against FZD3 or VANGL1 among various PCP proteins were effective for the LW histochemical staining. Note that the polarized distribution of FZD3 or VANGL1 proteins was not disturbed by *ANKS1A* deficiency (3 mice for each group). Scale bar, 5 [illegible]m. (e) Strategy in which *ANKS1A*-targeted cells were genetically labeled with a reporter (YFP) through induction of Cre recombinase activity. The floxed *ANKS1A* allele contained two loxP sites flanking three exons of *ANKS1A*, whereas the floxed *ROSA* allele contained two loxP sites flanking the *STOP* sequence. An *ANKS1A* BAC transgenic line expressing *CreER* was used to excise the floxed sequences in response to tamoxifen (TM) only in cells where *ANKS1A* is expressed. (f) TM was injected twice in total (on days P5 and P6). Then, the targeted E1 cells in the LW were analyzed at P15. The sections were stained with GT335 to visualize both targeted and non-targeted E1 cells. Scale bar, 5 μm. (g) The number of BBs was measured essentially as described in Supplementary Figure 2c. Control set, n = 113 cells from 2 mice; *ANKS1A* iKO, n = 87 cells from 2 mice.

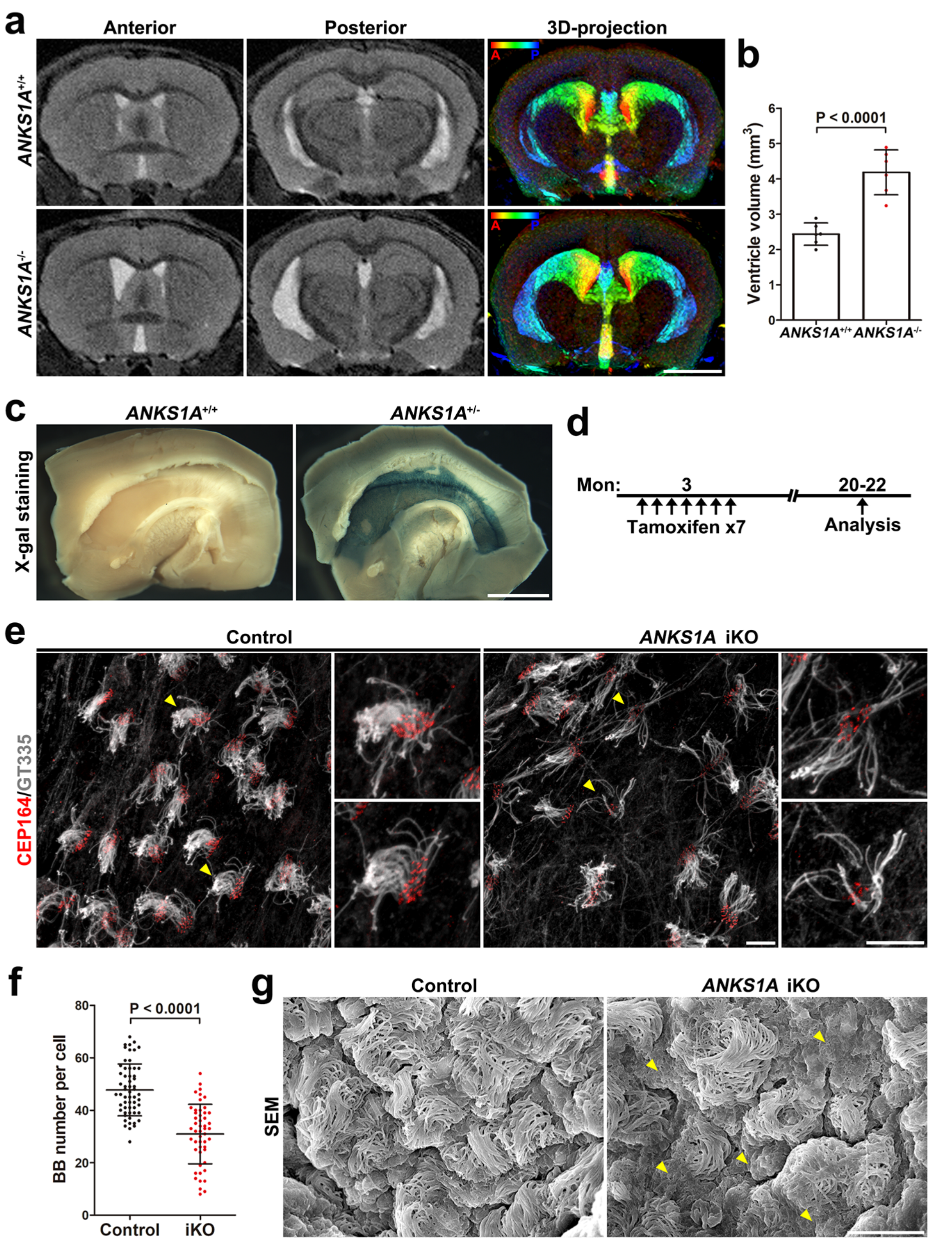

Supplementary Figure 5. (a) Brain ventricles were visualized using μMRI. Ventricles appeared bright on T2-weighted μMRI mouse brain images are shown in gray scale and color LUT display. Color-coded images display brain ventricles with anterior-posterior (A-P) direction. Red colors represent an anterior region of the brain ventricle, while blue colors represent a posterior region. Twenty consecutive coronal images were merged into a single picture for each brain (see supplementary Movie 4). Scale bar, 50 μm. (b) Ventricular volume in each brain was quantified using the Fiji image analysis package. *ANKS1A*$^{+/+}$ set, n = 6 mice; *ANKS1A*$^{-/-}$, n = 6 mice. (c) LWs with other forebrain tissues were isolated from WT or *ANKS1A*$^{+/lacZ}$ mice at three months of age and subjected to X-gal staining for 15 hours. Scale bar, 500 μm. (d) TM was injected once every day for a total of 7 consecutive days from mice at 3 months of age and the LWs from aged mice were isolated. (e) The LWs were stained with GT335 and CEP164 to visualize multi-cilia and BBs, respectively. Scale bar, 10 μm. (f) The number of BBs was measured essentially as described in Supplementary Figure 2f. Control set, n = 58 cells from 3 mice; *ANKS1A* iKO, n = 50 cells from 3 mice. (g) SEM analysis of LWs from littermates at 20 months of age (3 mice from each group). The yellow arrowheads mark the ependymal cells lacking multi-cilia. Scale bar,10 μm.

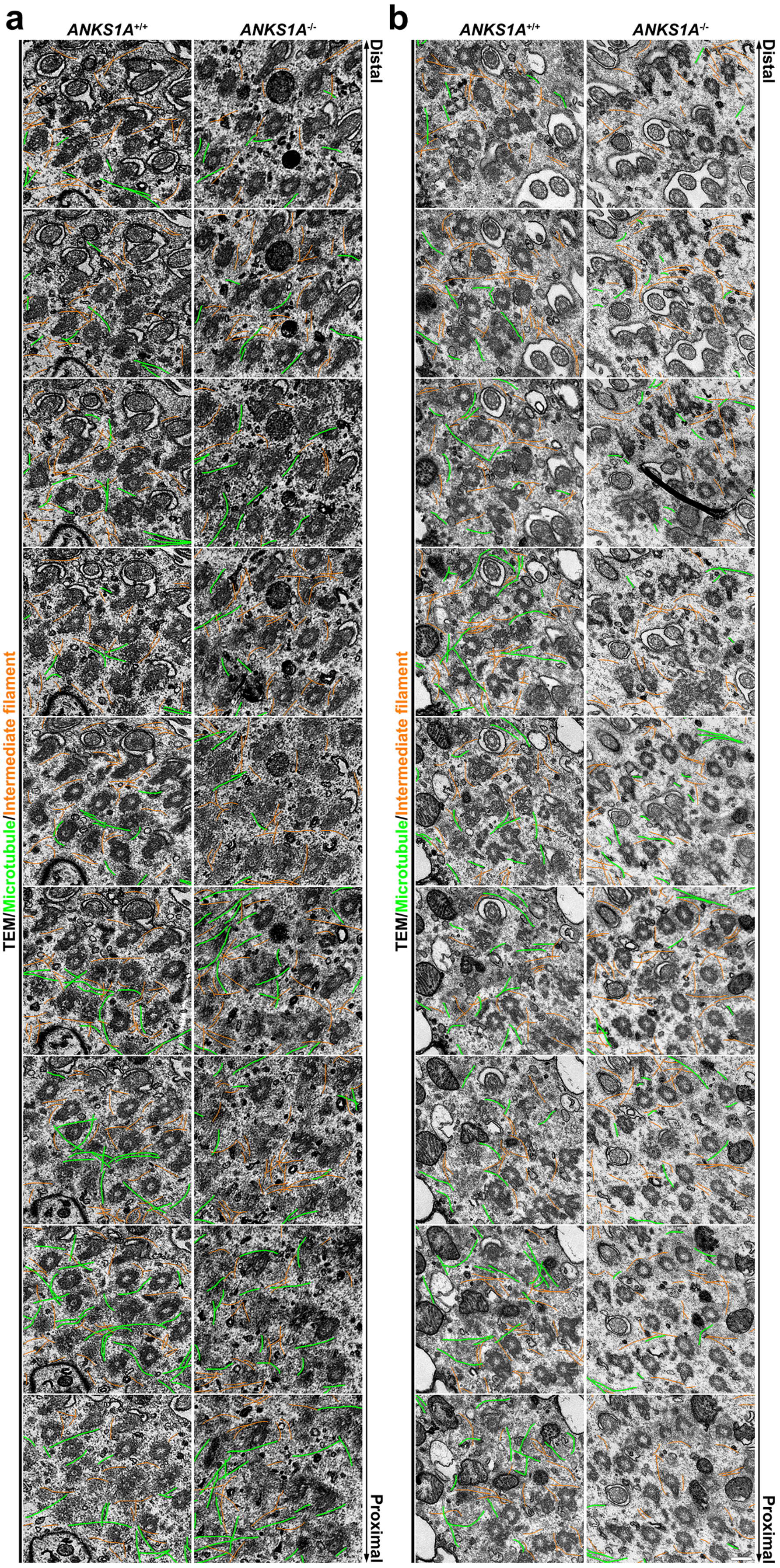
a
ANKS1A+/+
ANKS1A-/-
b
ANKS1A+/+
ANKS1A-/-
Distal
Proximal
TEM/Microtubule/Intermediate filament

Supplementary Figure 6. (a and b) A ribbon of consecutive sections (50 nm) was placed on a one-hole grid and subsequently analyzed by TEM. The three-dimensional organization of microtubules or intermediate filaments was examined 450 nm region beneath the initial portion of BF. Each image was enlarged up to 5X to visualize and draw the cytoskeletal networks and then each image was imported into the Zeiss Zen Blue software to reconstruct a 3D-image as shown in Fig. 7 and Movie 5. Scale bar, 500 nm.

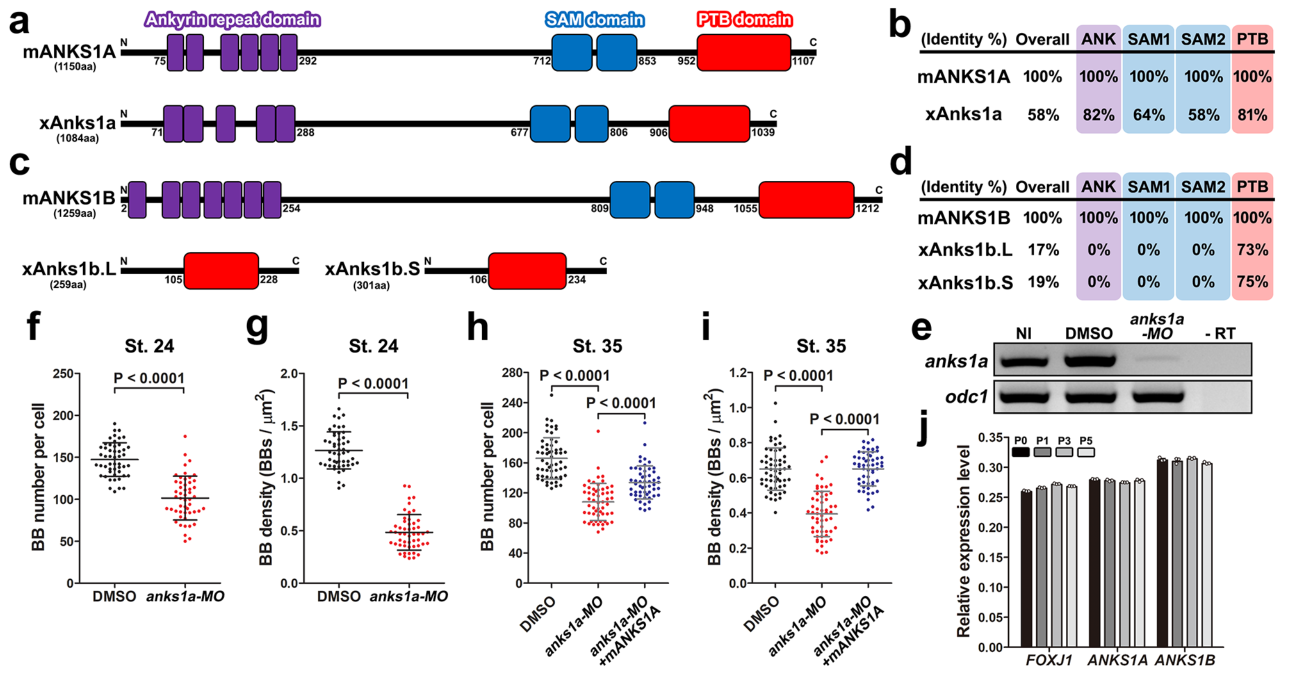

Supplementary Figure 7. (a-d) Schematic depicting the mouse and *Xenopus anks1* family gene homologues. A comparison of the amino acid sequences of the full-length proteins is shown with their conserved motifs. Note that the ankyrin repeats and PTB domain of ANKS1A are highly conserved with 82% and 81% identity, respectively. In contrast, the ankyrin repeats and SAM domains of mouse ANKS1B are not found in *Xenopus* Anks1B. The accession numbers of the genes used for these alignments were P59672 (m*ANKS1A*), Q6IRM7 (*x anks1a*), Q8BIZ1 (m*ANKS1B*), A0A1L8GWL7 (*xanks1b.L*) and A0A1L8GQZ5 (*xanks1b.S*). (e) RT-PCR analysis of *Xenopus anks1a* expression in animal cap explants isolated from embryos injected with DMSO or *anks1a*-MO. NI, not injected; *odc1*, *ornithine decarboxylase 1*. (f-i) Epidermis from *Xenopus* embryos at stage 24 or 35 was stained with anti-FOP antibodies and analyzed by 3DSIM analysis as described in Supplementary Figure 2c. For embryos at stage 24 (f & g), DMSO-injection, n = 50 cells from 3 embryos; *anks1a*-MO injection, n = 51 cells from 3 embryos. For embryos at stage 35 (h & i), DMSO-injection, n = 57 cells from 5 embryos; *anks1a*-MO injection, n = 56 cells from 5 embryos; *anks1a*-MO injection plus mouse *ANKS1A-VN* RNA injection, n = 54 cells from 3 embryos. (j) qRT-PCR analysis using total RNAs extracted from the LWs of mouse pups at the indicated postnatal stages. Note that both *ANKS1A* and *ANKS1B* are expressed in ependymal cells.
