# Supplementary material for: The molecular dynamics of subdistal appendages in multi-ciliated cells
Source: Nat Commun. 2021 Jan 27;12:612. doi: 10.1038/s41467-021-20902-4 (PMC7840914; doi:10.1038/s41467-021-20902-4)
Supplement: Supplementary file 3 — Description of Additional Supplementary Files [file 41467_2021_20902_MOESM3_ESM.docx]

**Description of Additional Supplementary Files**

File Name: Supplementary Movie 1

Description: Supplementary Movie 1 shows the real-time flow of fluorescent beads on live LW tissues in *ANKS1A*^+/+^ and *ANKS1A* ^-/-^ mice. The live LWs were prepared from young adult mice. Wild-type shows a typical AD-AV flow pattern, whereas KO does an abnormal and congested bead flow pattern with a 7-fold speed reduction.

File Name: Supplementary Movie 2

Description: Supplementary Movie 2 shows the 3D reconstruction of confocal images of YFP-labeled E1 cells in control and *ANKS1A* iKO mice. Tamoxifen was injected twice in total and then the targeted E1 cells were analyzed at P15. The sections were stained with GT335 (red) to visualize both targeted and non-targeted E1 cells. The cilia of YFP-labeled cells in the iKO samples show a scattered rather than bundled pattern, whereas this abnormality is not observed in YFP-negative neighboring cells.

File Name: Supplementary Movie 3

Description: Supplementary Movie 3 shows the real-time flow of fluorescent beads on live LW tissues in control and *ANKS1A* iKO mice. Tamoxifen was injected five times in total and then high-speed video imaging analysis was performed. Control shows a typical AD-AV flow pattern, whereas iKO does an abnormal and congested bead flow pattern with a 0.7-fold speed reduction.

File Name: Supplementary Movie 4

Description: Supplementary Movie 4 shows the 3D reconstruction of mouse brain μMRI images in gerontic *ANKS1A* ^+/+^ and *ANKS1A* ^-/-^ mice. Twenty consecutive coronal images were merged into a single picture for each brain. Red colors represent an anterior region of the brain ventricle, while blue colors a posterior region. This analysis shows that the *ANKS1A* KO brains have enlarged lateral ventricles.

File Name: Supplementary Movie 5

Description: Supplementary Movie 5 shows the 3D reconstruction of microtubule networks in gerontic *ANKS1A* ^+/+^ and *ANKS1A* ^-/-^ mice. The three-dimensional organization of microtubules (green) was examined 450 nm region beneath the initial portion of basal foot. This analysis shows that the aged KO mice have a significant reduction in the extent of microtubule networks in regions where basal bodies are defective.
