# Supplementary material for: The molecular dynamics of subdistal appendages in multi-ciliated cells
Source: Nat Commun. 2021 Jan 27;12:612. doi: 10.1038/s41467-021-20902-4 (PMC7840914; doi:10.1038/s41467-021-20902-4)
Supplement: Supplementary file 9 — Reporting Summary [file 41467_2021_20902_MOESM9_ESM.pdf]

nature research

Corresponding author(s): Soochul Park

Last updated by author(s): Sep 8, 2020

# Reporting Summary

Nature Research wishes to improve the reproducibility of the work that we publish. This form provides structure for consistency and transparency in reporting. For further information on Nature Research policies, see Authors & Referees and the Editorial Policy Checklist.

## Statistics

For all statistical analyses, confirm that the following items are present in the figure legend, table legend, main text, or Methods section.

| n/a | Confirmed | |
|---|---|---|
| ☐ | ☒ | The exact sample size ($n$) for each experimental group/condition, given as a discrete number and unit of measurement |
| ☐ | ☒ | A statement on whether measurements were taken from distinct samples or whether the same sample was measured repeatedly |
| ☐ | ☒ | The statistical test(s) used AND whether they are one- or two-sided<br>*Only common tests should be described solely by name; describe more complex techniques in the Methods section.* |
| ☒ | ☐ | A description of all covariates tested |
| ☐ | ☒ | A description of any assumptions or corrections, such as tests of normality and adjustment for multiple comparisons |
| ☐ | ☒ | A full description of the statistical parameters including central tendency (e.g. means) or other basic estimates (e.g. regression coefficient) AND variation (e.g. standard deviation) or associated estimates of uncertainty (e.g. confidence intervals) |
| ☐ | ☒ | For null hypothesis testing, the test statistic (e.g. $F$, $t$, $r$) with confidence intervals, effect sizes, degrees of freedom and $P$ value noted<br>*Give P values as exact values whenever suitable.* |
| ☒ | ☐ | For Bayesian analysis, information on the choice of priors and Markov chain Monte Carlo settings |
| ☒ | ☐ | For hierarchical and complex designs, identification of the appropriate level for tests and full reporting of outcomes |
| ☒ | ☐ | Estimates of effect sizes (e.g. Cohen's $d$, Pearson's $r$), indicating how they were calculated |

*Our web collection on statistics for biologists contains articles on many of the points above.*

## Software and code

Policy information about availability of computer code

| | |
|---|---|
| Data collection | LSM700 (Carl Zeiss Microscopy), ELYRA PS.1 (Carl Zeiss Microscopy), Axio Zoom.V16 (Carl Zeiss Microscopy), JSM-7600F (JEOL) field emission scanning electron microscopy (FE-SEM), JEM1010 (JEOL) transmission electron microscopy (TEM), Talos L120C (FEI) tranmission electron microscopy (TEM), MRI was conducted using a 9.4 T horizontal-bore Bruker Avance III HD imaging system (Bruker Biospin) |
| Data analysis | ZEN black (8.1) software, ZEN blue (1.1.2.0) software, ImageJ (Fiji) software, IMARIS (8.0.2) software, AngioTool (0.6 alpha) software, GraphPad Prism (5.01), SigmaPlot (7.0). |

For manuscripts utilizing custom algorithms or software that are central to the research but not yet described in published literature, software must be made available to editors/reviewers. We strongly encourage code deposition in a community repository (e.g. GitHub). See the Nature Research guidelines for submitting code & software for further information.

## Data

Policy information about availability of data

All manuscripts must include a data availability statement. This statement should provide the following information, where applicable:

- Accession codes, unique identifiers, or web links for publicly available datasets
- A list of figures that have associated raw data
- A description of any restrictions on data availability

All data supporting the findings of this study are available from the corresponding author upon reasonable request.

# Field-specific reporting

Please select the one below that is the best fit for your research. If you are not sure, read the appropriate sections before making your selection.

☒ Life sciences ☐ Behavioural & social sciences ☐ Ecological, evolutionary & environmental sciences

For a reference copy of the document with all sections, see nature.com/documents/nr-reporting-summary-flat.pdf

# Life sciences study design

All studies must disclose on these points even when the disclosure is negative.

| | |
|---|---|
| Sample size | ~~Sample size was determined to be based on the magnitude and consistency of measurable differences between groups. For statistical significance, at least three independent experiments were subjected.~~ |
| Data exclusions | No data were excluded. |
| Replication | Each experiment presented in the paper was repeated in multiple times and/or across multiple animals. Replicate experiments were successful. The precise number of repeats are given in the figure legend. |
| Randomization | We did not use randomization to assign animals to experimental groups. Mice analyzed were litter mates and sex-matched whenever possible. |
| Blinding | All investigators were blinded to group allocation during data collection and analysis. |

# Reporting for specific materials, systems and methods

We require information from authors about some types of materials, experimental systems and methods used in many studies. Here, indicate whether each material, system or method listed is relevant to your study. If you are not sure if a list item applies to your research, read the appropriate section before selecting a response.

## Materials & experimental systems

| n/a | Involved in the study | |
|---|---|---|
| ☐ | ☒ | Antibodies |
| ☒ | ☐ | Eukaryotic cell lines |
| ☒ | ☐ | Palaeontology |
| ☐ | ☒ | Animals and other organisms |
| ☒ | ☐ | Human research participants |
| ☒ | ☐ | Clinical data |

## Methods

| n/a | Involved in the study | |
|---|---|---|
| ☒ | ☐ | ChIP-seq |
| ☒ | ☐ | Flow cytometry |
| ☐ | ☒ | MRI-based neuroimaging |

## Antibodies

| | |
|---|---|
| Antibodies used | The following antibodies were used: rabbit IgG anti-Anks1a (1:100, Bethyl, A303-049A), rabbit IgG anti-Anks1a (1:100, Bethyl, A303-050A), mouse IgG1 anti-CNTRL (1:100, Santa Cruz, sc-365521), mouse IgG2b anti-FOP (1:250, Abnova, H00011116-M01), rabbit IgG anti-CEP164 (1:200, Atlas antibodies, HPA037606), mouse IgG2a anti-Centrin (1:100, Millipore, 04-1624), rabbit IgG anti-ODF2 (1:100, Atlas antibodies, HPA001874), mouse IgG1 anti-GT335 (1:200, Adipogen, AG-20B-0020), mouse IgG2b anti-Ac-tubulin (1:200, Sigma Aldrich, T7451), mouse IgG1 anti-γ-tubulin (1:100, Abcam, ab11316), phalloidin-Atto647N anti-F-actin (1:50, Sigma Aldrich, 65906), mouse IgG1 anti-ZO-1-488 (1:100, Invitrogen, 339188), chick anti-GFP (1:250, Abcam, ab13970). rabbit anti-CEP19 (1:100, Proteintech, 26036-1-AP), mouse IgG2a anti-a-tubulin (1:200, Santa Cruz, SC5286), mouse IgG2a anti-Frizzled 3 (1:100, Sigma Aldrich, SH0007976M9), rabbit anti-Vangl1 (1:100, Atlas antibodies, HPA025235), rabbit anti-CEP350 (1:1000, Novus, NB100-59811). |
| Validation | ~~Anks1a antibody was validated in Anks1a KO mice from our group.~~<br>~~FOP, CEP19 antibodies were validated in each KO hTERT-RPE cells. (Reference: PubMed: 28625565)~~<br>~~ODF2 antibody was validated in ODF2 KO mice. (Reference: PubMed: 22265411)~~<br>~~When available, we purchased commercial antibodies that have been previously validated in multiple independent studies.~~ |

## Animals and other organisms

Policy information about studies involving animals; ARRIVE guidelines recommended for reporting animal research

| | |
|---|---|
| Laboratory animals | Laboratory mice were maintained on a congenic C57BL/6 background. Both male and female mice were used in this study. Mice were sacrificed at P0-P2 for ependymal cell cultures. MRI analysis was performed on old mice of 18-22 months of age. Anks1a+/lacZ gene trap mice have been previously described, (Kim, J. et al., 2010). |

| | |
|---|---|
| | Anks1a-CreER mice were generated in this paper using the same approach as we previously described, (Park, S. et al., 2019). R26-stop-ETFP mice were purchased from The Jackson Laboratory, RPID: IMSR_JAX:006148 Anks1af/+ mice were generated in this paper. Xenopus laevis (females for eggs collection and males for sperm collection) from the Jaebong's lab in Hallym university. |
| Wild animals | N/A |
| Field-collected samples | N/A |
| Ethics oversight | All experiments were approved by and were in compliance with the Sookmyung Women's University Institutional Animal Care and Use Committee (SWU-IACUC-2001-029). All mice were housed and handled at the animal facility of Sookmyung Women's University. Xenopus embryo study was conducted in accordance with the regulations of the Institutional Animal Care and Use Committees (IACUC) of Hallym University (Hallym2019-81). All the research members attended both the educational and training courses for the appropriate care and use of experimental animals at our institutions in order to receive an animal use permit. |

Note that full information on the approval of the study protocol must also be provided in the manuscript.

# Magnetic resonance imaging

## Experimental design

| | |
|---|---|
| Design type | *Indicate task or resting state; event-related or block design.* |
| Design specifications | *Specify the number of blocks, trials or experimental units per session and/or subject, and specify the length of each trial or block (if trials are blocked) and interval between trials.* |
| Behavioral performance measures | *State number and/or type of variables recorded (e.g. correct button press, response time) and what statistics were used to establish that the subjects were performing the task as expected (e.g. mean, range, and/or standard deviation across subjects).* |

## Acquisition

| | |
|---|---|
| Imaging type(s) | Structural MRI |
| Field strength | 9.4T |
| Sequence & imaging parameters | Turbo spin echo sequence.<br>Coronal: field of view(FOV) 20 x 20 mm, matrix 256 x 256, TR/TE = 4300/26 ms, slice thickness 0.25 mm |
| Area of acquisition | Whole-brain scan was performed, followed by volume analysis of brain ventricle. |

Diffusion MRI ☐ Used ☒ Not used

## Preprocessing

| | |
|---|---|
| Preprocessing software | *Provide detail on software version and revision number and on specific parameters (model/functions, brain extraction, segmentation, smoothing kernel size, etc.).* |
| Normalization | *If data were normalized/standardized, describe the approach(es): specify linear or non-linear and define image types used for transformation OR indicate that data were not normalized and explain rationale for lack of normalization.* |
| Normalization template | *Describe the template used for normalization/transformation, specifying subject space or group standardized space (e.g. original Talairach, MNI305, ICBM152) OR indicate that the data were not normalized.* |
| Noise and artifact removal | *Describe your procedure(s) for artifact and structured noise removal, specifying motion parameters, tissue signals and physiological signals (heart rate, respiration).* |
| Volume censoring | *Define your software and/or method and criteria for volume censoring, and state the extent of such censoring.* |

## Statistical modeling & inference

| | |
|---|---|
| Model type and settings | *Specify type (mass univariate, multivariate, RSA, predictive, etc.) and describe essential details of the model at the first and second levels (e.g. fixed, random or mixed effects; drift or auto-correlation).* |
| Effect(s) tested | *Define precise effect in terms of the task or stimulus conditions instead of psychological concepts and indicate whether ANOVA or factorial designs were used.* |

Specify type of analysis: ☐ Whole brain ☐ ROI-based ☐ Both

| | |
|---|---|
| Statistic type for inference (See Eklund et al. 2016) | *Specify voxel-wise or cluster-wise and report all relevant parameters for cluster-wise methods.* |

Correction

*Describe the type of correction and how it is obtained for multiple comparisons (e.g. FWE, FDR, permutation or Monte Carlo).*

## Models & analysis

| n/a | Involved in the study |
| --- | --- |
| ☒ | ☐ Functional and/or effective connectivity |
| ☒ | ☐ Graph analysis |
| ☒ | ☐ Multivariate modeling or predictive analysis |
